# Biocidal Activity of Selected Essential Oils Against Bacterial and Fungal Pathogens and Their Cytotoxicity Toward Human Cell Lines

**DOI:** 10.3390/microorganisms14102196

**Published:** 2026-10-01

**Authors:** Antoni Woźniak, Kamil Drożdż, Katarzyna Biegun-Drożdż, Tomasz Gosiewski, Tomasz Cudak, Robert Karpiński, Aleksandra Fiołek, Aleksandra Błoniarz, Jakub Marchewka, Tomasz Moskalewicz, Monika Brzychczy-Włoch

**Affiliations:** 1 Department of Molecular Medical Microbiology, Faculty of Medicine, Medical College, Jagiellonian University, Czysta 18, 31-008 Kraków, Poland; antekwozniak703@gmail.com (A.W.); kamil.drozdz@uj.edu.pl (K.D.); ctomasz.gosiewski@uj.edu.pl (T.G.); 2 Faculty of Metals Engineering and Industrial Computer Science, AGH University of Krakow, Av. A. Mickie-wicza 30, 30-059 Kraków, Poland; tcudak@agh.edu.pl (T.C.); rkarpinski@agh.edu.pl (R.K.); kruka@agh.edu.pl (A.F.); debovska@agh.edu.pl (A.B.); tmoskale@agh.edu.pl (T.M.); 3 Faculty of Materials Science and Ceramics, AGH University of Krakow, Av. A. Mickiewicza 30, 30-059 Kraków, Poland; jmar@agh.edu.pl

**Keywords:** essential oils, antimicrobial resistance, biocidal activity, minimum bactericidal concentration, cytotoxicity, MTT assay

## Abstract

The growing global burden of antimicrobial resistance (AMR) highlights the urgency of identifying new therapeutic options or adjuvant agents capable of limiting microbial growth. This study aimed to evaluate the potential applicability of selected essential oils (EOs) as alternative antimicrobial agents by assessing their biocidal activity against clinically relevant microorganisms and determining their cytotoxicity toward human cell lines. Serial dilutions of the studied EOs were prepared to determine the minimum bactericidal (MBC), minimum fungicidal (MFC), and minimum biofilm eradication concentrations (MBEC) against *Staphylococcus aureus* ATCC^®^ 25923, *Escherichia coli* ATCC^®^ 25922, *Candida albicans* ATCC^®^ 90028, and *Nakaseomyces glabratus* ATCC^®^ 15545. Cytotoxicity towards cell lines was evaluated using the MTT assay. The tested EOs displayed significant biocidal effects against studied microorganisms in both planktonic and biofilm forms. Cinnamon bark oil and cinnamaldehyde showed the highest biocidal activity against *S. aureus* ATCC^®^ 25923, *E. coli* ATCC^®^ 25922, *C. albicans* ATCC^®^ 90028, and *N. glabratus* ATCC^®^ 15545, except for *S. aureus* in biofilm form. For thymol, yeast strains exhibited eight-fold lower susceptibility compared to bacterial strains. Cytotoxicity of the tested EOs towards the studied cell lines was significant. EOs proved to be effective against microorganisms but at the same time showed substantial cytotoxicity, thus potentially limiting their application. Further research on formulation strategies, selective delivery, and dose optimization is required to enhance their safety profile and clinical feasibility.

## 1. Introduction

Essential oils (EOs) represent a promising group of compounds that may enhance conventional antibiotic therapies. EOs are plant-derived secondary metabolites, which can be isolated from various plant tissues, including bark, buds, and flowers, where they can act as an antimicrobial, an insecticide, and a repellent against herbivores. EOs consist of various chemical compounds, the majority of which are of two distinct biosynthetic origins [1]. There are five distinct groups: terpenoids, aliphatic compounds, aromatic compounds, nitrogen-containing compounds, and sulfur-containing compounds. Terpenoids can be further divided into subgroups based on the presence or absence of oxygen and the number of isoprenoid units, into mono, sesqui, di and tri terpenes. Monoterpenes and sesquiterpenes are the most abundant representative molecules in EOs, belonging to the terpene group, with isoprene (C5) being the basic structural unit. Terpenoids include alcohols, aldehydes, acids, phenols, ethers, and esters. EOs may also contain phenylpropanoids, a class of aromatic compounds that, although generally less abundant, are predominant in certain species such as *Cinnamomum verum*, where cinnamaldehyde is the major constituent [2].

EOs are generally volatile and soluble in organic solvents. The lipophilic properties of EOs are strictly connected to their commonly postulated antimicrobial modes of action, including cell membrane disruption, membrane permeabilization, impairment of energy metabolism, induction of oxidative stress, and interference with quorum sensing and biofilm formation [3]. The potential use of EOs either alone or in combination with conventional antibiotic treatment may be a way of addressing the rise in AMR [4]. In such an approach, the assessment of the cytotoxicity of the compounds used is crucial, especially when considering the postulated mode of action of EOs.

As the characteristics of EOs have been widely described in the literature, some aspects of potential therapeutic use still need further research. First, antimicrobial activity, antibiofilm activity and cytotoxicity of EOs are often studied independently in separate studies, using different compounds, their concentrations, and different microbial strains. Such an approach precludes the evaluation of the direct relationship between the concentration required to elicit the antimicrobial activity and cytotoxic concentrations in human cell lines. Second, it is often not clear if the observed antimicrobial activity is due to the activity of the predominant compound of an EO or if the antimicrobial activity is the result of interactions among multiple constituents, as whole EOs and their constituents are rarely studied together under the same conditions. Third, there are insufficient data on the susceptibility of some clinically important strains, such as *N. glabratus*.

This study aimed to assess the biocidal effect of EOs and their isolated components against *S. aureus* ATCC^®^ 25923, *E. coli* ATCC^®^ 25922, *C. albicans* ATCC^®^ 90028, and *N. glabratus* ATCC^®^ 15545, in both planktonic and biofilm forms, as well as to evaluate their cytotoxicity toward A549, MG-63 and FaDu cell lines. Assessing both microbial forms provides a more comprehensive understanding of the antimicrobial potential of EOs and is particularly relevant due to the enhanced resilience and eradication challenges associated with biofilm. We hypothesized that selected EOs would display significant biocidal activity against the studied microorganisms while presenting non-significant cytotoxicity towards the tested cell lines, thus indicating their potential as a safe and effective alternative to conventional treatments.

## 2. Materials and Methods

### 2.1. Selected Essential Oils and Their Major Constituents

In this study, the following EOs and their major components were used (Table 1). The main components of the essential oils studied were selected based on a critical review of the literature and the manufacturers’ specifications, considering both their quantitative composition in a given oil and the antimicrobial activity attributed to them in the literature.

All the studied EOs were stored in dark-brown glass bottles at room temperature, protected from light.

All essential oils and their components were characterized using FT-IR spectroscopy with a Bruker Vertex 70v spectrometer (Bruker, Billerica, MA, USA). For liquids (all substances except thymol), the attenuated total reflection (ATR) technique and zinc selenide attachment (Buck Scientific, East Norwalk, CT, USA) were employed. For thymol, the standard KBr pellet technique was used. The measurements were performed in the range of 550–4000 cm^−1^ with a resolution of 4 cm^−1^, collecting 128 scans. OPUS 7.2 software (Bruker, Billerica, MA, USA) was used for data processing. FT-IR spectroscopy was used to confirm the identity of the substances and to verify that the spectra of the essential oils matched those of their main components.

### 2.2. Antimicrobial Activity of Essential Oils Against Bacterial and Yeast Strains

#### 2.2.1. Microbial Cultures

Microbial cultures of *S. aureus* ATCC^®^ 25923, *E. coli* ATCC^®^ 25922, *C. albicans* ATCC^®^ 90028, and *N. glabratus* ATCC^®^ 15545 were obtained from the American Type Culture Collection (ATCC, Manassas, VA, USA). *S. aureus* and *E. coli* were inoculated onto tryptic soy broth (TSB) (Becton Dickinson, Franklin Lakes, NJ, USA) and incubated at 37 °C for 18 h. *C. albicans* and *N. glabratus* were cultured in Sabouraud broth (SB) (Liofilchem, Roseto degli Abruzzi, Italy) and incubated at 37 °C for 48 h.

These strains represent microorganisms with distinct cell-envelope structures (Gram-positive, Gram-negative bacteria, and yeast) and biofilm-forming capacities, providing a clinically relevant model for evaluating the biocidal activity of EOs.

#### 2.2.2. Essential Oils Serial Dilutions

To prepare serial dilutions, 1% (*v*/*v*) of Tween 20 (Sigma-Aldrich, St. Louis, MO, USA) was added to sterile TSB and SB, 20 mL of each medium was taken prior to act as a blank. For the majority of the studied EOs, concentrations ranged from 5% to 0.0625%, with 100% representing the stock solution supplied by the manufacturer (Table 1). For CiO-JT on *E. coli*, the concentration range was increased to 30% as lower concentrations did not yield an MBC value. A volume of 2 mL of solution was taken from each concentration into two separate eppendorf tubes (1 mL each). Cultured bacteria (18 h) and yeast (48 h) were suspended in previously prepared media (TSB and SB) with 1% (*v*/*v*) Tween 20 to obtain a concentration of 10^4^ CFU/mL (colony-forming units). Both media contained 1% (*v*/*v*) Tween 20 to match the concentration in EO serial dilutions.

#### 2.2.3. Minimum Bactericidal Concentration Assay

To establish the minimum bactericidal (MBC) and fungicidal (MFC) concentrations of the EOs, the final bacterial and yeast inoculum was adjusted to 1 × 10^4^ CFU/mL, based on the broth microdilution approach recommended in the EUCAST reading guide (v 4.0, 1 January 2022) [5].

Subsequently, standardized suspensions of *S. aureus*, *E. coli*, *C. albicans* and, *N. glabratus* were added to the EO serial dilutions to obtain a final inoculum of 10^4^ CFU/mL. Prepared samples in triplicate (n = 3) were incubated at 37 °C for 18 h, followed by culturing on blood agar (Oxoid Ltd., Basingstoke, UK) and Sabouraud agar (Liofilchem, Roseto degli Abruzzi, Italy) for bacterial and yeast strains, respectively. Positive controls (n = 3) included the media without the studied compounds. After incubation, the plates were evaluated. The MBC and MFC for each EO were set based on plates of the lowest EO concentration where no cultures were observed (Figure 1).

#### 2.2.4. Minimum Biofilm Eradication Concentration Assay

To establish the minimum biofilm eradication concentrations (MBEC), the MBEC_50_ and MBEC_90_ values were determined. MBEC_50_ was defined as the lowest concentration of an EO that eradicated ≥50% of the biofilm, whereas MBEC_90_ corresponded to ≥90% eradication. CL-MIX, TTO, CB, CA, and CV were selected as representatives of the studied EO groups. Two representatives from the cinnamon group were included based on their previously obtained MBC/MFC values. Biofilm eradication was assessed by measuring the metabolic activity of microorganisms using the resazurin reduction assay. Elements of our methodological strategy were inspired by the framework proposed by [6], which we adapted to suit our experimental design. Bacterial and fungal suspensions were prepared in TSB and SB, respectively, and adjusted to 0.5 McFarland standard. A volume of 1 mL of the suspensions was inoculated into 24-well (Nest Biotechnology Co., Ltd., Wuxi, China) plates and incubated at 37 °C for 24 h. Subsequently, the medium was replaced with 1 mL of the prepared dilution series of the selected EOs, and the plates were incubated under the same conditions. Each dilution was tested in three independent wells (n = 3). Positive controls included wells (n = 3) without the studied compounds. After incubation, the EO solutions were removed, and wells were gently washed with phosphate-buffered saline (PBS) to remove planktonic cells.

To measure the metabolic activity, 1 mL of fresh TSB/SB with 10% AlamarBlue reagent (Sigma-Aldrich, St. Louis, MO, USA) was added to each well and incubated at 37 °C for 20 min. Positive control included the reagent reduced at 100 °C for 20 min. Then, 100 µL of the supernatant was placed in triplicate in a black 96-well plate (Nunc, Thermo Fisher Scientific, Waltham, MA, USA). Fluorescence was measured using a SpectraMax^®^ Mini plate reader (Molecular Devices, San Jose, CA, USA) at excitation/emission wavelengths of 544/590 nm.

### 2.3. In Vitro Cytotoxicity

#### 2.3.1. Cell Line Cultures

Cell viability assays were performed using human cell lines FaDu (ATCC^®^ HTB-43), MG-63 (ATCC^®^ CRL-1427), and A549 (ATCC^®^ CCL-185) obtained from the American Type Culture Collection. FaDu and MG-63 cells were cultured in Eagle’s Minimum Essential Medium (EMEM) (Gibco, Thermo Fisher Scientific, Paisley, UK), whereas A549 cells were maintained in Dulbecco’s Modified Eagle Medium (DMEM) (Gibco, Thermo Fisher Scientific, Paisley, UK). All media were supplemented with 10% fetal bovine serum (Gibco, Thermo Fisher Scientific, Paisley, UK) and the antibiotic solution (ZellShield^®^, Minerva Biolabs, Berlin, Germany). Cells were grown in standard tissue culture flasks (Sarstedt, Nümbrecht, Germany) at 37 °C in a humidified atmosphere containing 5% CO_2_.

#### 2.3.2. Preparation of Cell Cultures

Before the experiment, A549, MG-63, and FaDu cells were inoculated into 96-well plates (Nest Biotechnology Co., Ltd., Wuxi, China) at a density of 10,000 cells per well in 200 µL of the appropriate culture medium. Plates were incubated for 24 h at 37 °C and 5% CO_2_ to allow for cell adhesion and stabilization.

#### 2.3.3. Exposure of Cells to Essential Oils

On the following day, a series of dilutions of the tested essential oils was prepared in the range of 0.5% to 0.004% (*v*/*v*) using the appropriate culture medium, with 100% representing the stock solution supplied by the manufacturer (Table 1). After culturing, the medium was removed from each well, and 200 µL of fresh medium containing the EO dilutions was added. Cells were then incubated for an additional 24 h at 37 °C and 5% CO_2_ (Figure 2).

#### 2.3.4. MTT Assay

After the 24-h exposure to EOs, 20 µL of 3-(4,5-dimethylthiazol-2-yl)-2,5-diphenyltetrazolium bromide (MTT) solution (Sigma-Aldrich, St. Louis, MO, USA) was added to each well at a concentration of 5 mg/mL. Plates were incubated for 3 h at 37 °C and 5% CO_2_. Following incubation, the medium was removed, and the resulting formazan crystals were dissolved by adding 200 µL of dimethyl sulfoxide (DMSO) (VWR International, Gdańsk, Poland) to each well. The solubilized formazan was then transferred in 100 µL aliquots to a new 96-well plate (Nest Biotechnology Co., Ltd., Wuxi, China) for spectrophotometric measurement. Absorbance was measured at λ = 563 nm using an Infinite^®^ M Nano microplate reader (Tecan, Männedorf, Switzerland). Each essential oil was tested in three independent replicates (n = 3). Control wells contained cells cultured in EO-free medium.

#### 2.3.5. Data Analysis

Relative cell viability (RV) was calculated using the following equation:

(1) $$\mathrm{RV}\;[\%]\;=\;\frac{A_{E}}{A_{C}}\;\times \;100\%$$

where A_E_ is the absorbance in wells treated with the essential oil, and A_C_ is the absorbance in control wells (cells not exposed to the EO).

Cytotoxicity was assessed by determining the impact of essential oils on cell survival in comparison with untreated control cells. In accordance with ISO 10993-5 [7], essential oils that reduced cell viability below 70% were interpreted as cytotoxic, whereas those for which cell viability remained at or above 70% were considered to exhibit no cytotoxic effect.

The selectivity index (SI) was calculated using the following equation:

$$SI\;=\;\frac{{CC}_{70}}{{MBC/MFC/MBEC}_{50}{/MBEC}_{90}}$$

where CC_70_ is the minimal concentration of an essential oil required to obtain the 70% cut-off value (according to ISO 10993-5), MBC is the minimum bactericidal concentration, MFC is the minimum fungicidal concentration, MBEC_50_ is the minimum biofilm eradication concentration defined as the lowest concentration of an EO that eradicated ≥50% of the biofilm, and MBEC_90_ is the minimum biofilm eradication concentration, defined as the lowest concentration of an EO that eradicated ≥90% of the biofilm.

## 3. Results

### 3.1. FT-IR Characterization of Essential Oils

The main components of citronella oil are citronellal, which is an unsaturated aldehyde, as well as geraniol and citronellol, both of which are unsaturated alcohols (Figure 3a–c). Substances referred to as citronellol (mixture of isomers), citronellol and β-citronellol are the same chemical compound, as the name citronellol (mixture of isomers) emphasizes its nature as a mixture of two stereoisomers with R-(+) and S-(−) configurations and the name β-citronellol underlines the specific position of the double bond in the molecule structure. This is confirmed by the FT-IR spectra obtained for these three substances (Figure 4b–d), which show bands with similar positions and intensities. Analysis of the spectrum for citronellol (a mixture of isomers) showed that the most intense band, located at 1057 cm^−1^ for C-O stretching, is typical of the presence of hydroxyl groups in alcohols. Also characteristic for them are the broad band at 3323 cm^−1^, corresponding to O–H stretching, and the band at 962 cm^−1^, corresponding to O–H bending. The bands at 1377 cm^−1^ and 830 cm^−1^ could be attributed to C–H bending in the -C=C-CH moiety. Also distinctive are the bands in the range of approximately 2855–2965 cm^−1^ for C–H stretching, as well as at 1452 cm^−1^ and 1010 cm^−1^ for C–H bending. The weak band at 1672 cm^−1^ could be assigned to C=C stretching in the structure of unsaturated compounds. In the FT-IR spectrum of citronella oil (Figure 4a), the most significant bands are those corresponding to functional characteristics of citronellal. Typical for the aldehyde structure are the most intense band at 1725 cm^−1^ for C=O stretching and the weak band at 2722 cm^−1^ for C–H stretching in the aldehyde group. The band at 3439 cm^−1^ for O–H stretching could be attributed to the presence of citronellol and geraniol. Other distinctive bands in the range of 2855–2965 cm^−1^ for C–H stretching, as well as at 1449 cm^−1^, 1378 cm^−1^, 1108 cm^−1^, 1015 cm^−1^ and 829 cm^−1^ for C–H bending, may reflect multiple constituents of citronellal oil. Similarly, the bands at 1671 cm^−1^ and 1643 cm^−1^ correspond to C–C stretching.

In the composition of tea tree oil, the main ingredients are terpinen-4-ol, γ-terpinene, and α-terpinene. Terpinen-4-ol is a tertiary alcohol with a relatively complex structure containing a substituted unsaturated six-membered ring (Figure 3d). In the FT-IR spectrum of this compound (Figure 4f), characteristic bands are those associated with the presence of the hydroxyl group at 3451 cm^−1^ for O–H stretching, 1302 cm^−1^ for O–H bending, and 1125 cm^−1^ for C–O bending. The bands in the range of approximately 2875–2965 cm^−1^ are attributed to C–H stretching in the methyl and methylene groups. The bands at 1469 cm^−1^, 1439 cm^−1^ and 799 cm^−1^ are associated with C–H bending in the C=C-CH3 and C=C-CH2 moieties, while the band at 1379 cm^−1^ is associated with C–H bending in the isopropyl substituent. Several bands could be attributed to C–C stretching. This includes the bands at 1250 cm^−1^ and 1225 cm^−1^ associated with the presence of the isopropyl substituent; those at 1180 cm^−1^ and 1160 cm^−1^ associated with the methyl substituent; and a series of bands corresponding to skeletal vibrations of the six-membered ring in the range of approximately 860–1070 cm^−1^, among which those at approximately 860–925 cm^−1^ correspond to C–C vibrations near the double bond. Finally, the weak band at 1678 cm^−1^ is typical of C=C stretching in unsaturated compounds. The FT-IR spectrum of tea tree oil (Figure 4e) is similar to that of terpinen-4-ol, as this compound is the main component of this oil. However, a few additional bands related to the presence of other ingredients, such as γ-terpinene and α-terpinene, could be observed. Weak bands at 1658 cm^−1^ and 1644 cm^−1^ for C=C stretching are characteristic of their unsaturated structure. Similarly, the bands at 825 cm^−1^ for C–C stretching and those at 782 cm^−1^ for C–H bending near the double bond are also typical.

The composition of cinnamon oils depends on their source. Cinnamon leaf oil mainly contains eugenol, while cinnamon bark oil is mainly composed of cinnamaldehyde, with a relatively significant proportion of eugenol. Eugenol is an aromatic phenol substituted with the methoxy and allyl groups (Figure 3e). In its FT-IR spectrum (Figure 4i), the most characteristic bands are located at 1231 cm^−1^ for O–H stretching typical of phenols, as well as at 1264 cm^−1^ and 1032 cm^−1^ for C–O–C stretching related to the presence of the methoxy group. The most intense band at 1510 cm^−1^ and the one at 1431 cm^−1^ are attributed to C=C stretching in the aromatic ring. A number of bands are associated with the other vibrations in the aromatic ring. In the range of approximately 1120–1210 cm^−1^, they are typical of C–H vibrations, and at 745 cm^−1^, 793 cm^−1^, 850 cm^−1^ and 912 cm^−1^, they are associated with C–H stretching. A weak band at 1638 cm^−1^ can be attributed to C=C stretching in the allyl substituent. Cinnamaldehyde is an α,β-unsaturated aromatic aldehyde (Figure 3f). In its FT-IR spectrum (Figure 4j) the most characteristic bands are present at 1667 cm^−1^ for C=O stretching and at 1118 cm^−1^ for C–O stretching in the aldehyde group. The band at 1623 cm^−1^ is typical of C=C stretching in the system conjugated with the aromatic ring. As in the case of eugenol, a number of bands are associated with the aromatic ring vibrations. The bands in the range of approximately 1450–1610 cm^−1^ are typical of C–C stretching, and the bands at 685 cm^−1^ and 744 cm^−1^ are related to C–H bending. In addition, distinctive bands for C–H bending associated with the presence of a double bond at 1294 cm^−1^ and 969 cm^−1^ are also present. The FT-IR spectrum of cinnamon bark oil (Figure 4h) shows features similar to the FT-IR spectrum of cinnamaldehyde. Additionally, a few weak bands characteristic of eugenol were observed. These are primarily the bands at 1270 cm^−1^ for C–O–C stretching in the methoxy group, which is the substituent of the aromatic ring, and at 1237 cm^−1^ for C–O stretching and 818 cm^−1^ for O–H bending typical of phenols. On the other hand, the FT-IR spectrum of cinnamon leaf oil (Figure 4g) closely resembles the FT-IR spectrum of eugenol.

Finally, in the composition of thyme oil, the main ingredients are thymol, γ-terpinene, and carvacrol. Thymol is a substituted aromatic phenol (Figure 3g). Therefore, the most characteristic bands in its FT-IR spectrum (Figure 4l) are those at 1422 cm^−1^ and 738 cm^−1^ for C–O bending and at 1244 cm^−1^ for C–O stretching in the hydroxyl group attached to the aromatic ring. In addition, the broad band at 3235 cm^−1^ for O–H stretching is typical. Here again, a number of bands could be identified for the aromatic ring vibrations.

The bands in the range of approximately 1460–1625 cm^−1^ are attributed to C–C stretching, and the band at 588 cm^−1^ is assigned to C–C bending. In addition, the bands at 806 cm^−1^ and 944 cm^−1^ are related to C–H bending. Since thymol has methyl and isopropyl substituents in its structure, some bands are associated with their vibrations. Typical ones are in the range of about 2865–2960 cm^−1^ for C–H stretching, at 1287 cm^−1^ for C–H bending, and at 1158 cm^−1^ and 1091 cm^−1^ for C–C bending. Carvacrol is an isomer of thymol (Figure 3h) and, for this reason, the FT-IR spectrum of this compound (Figure 4m) is similar to that of thymol. The differences arise from the presence of different intermolecular interactions, as carvacrol is a liquid and thymol is a crystalline solid. This is confirmed by the significant difference in the position of the band for O–H stretching, which is shifted towards higher wavenumbers to 3368 cm^−1^. The positions of the other bands are similar, although some differ in intensity. Also noticeable are the other bands at 1115 cm^−1^ and 864 cm^−1^, which are typical of C–H stretching, as well as the band at 638 cm^−1^ for C–C bending in the aromatic ring. The bands at 1173 cm^−1^ and 994 cm^−1^ could be attributed to C–C stretching in the isopropyl substituent. The FT-IR spectrum of thymol oil (Figure 4k) closely resembles that of thymol. Any bands associated with the presence of carvacrol and γ-terpinene are located in similar positions to the bands characteristic of thymol and therefore cannot be easily distinguished.

### 3.2. Antimicrobial Activity of EOs Against Bacterial and Yeast Strains

The results of antimicrobial activity are shown in Figure 5. The lowest values were obtained in the cinnamon group (0.063–1%). For bacterial strains in this group, MBC did not exceed 0.25%. The highest MBC was observed for Java-type citronella oil (CIO-JT) on *E. coli* ATCC^®^ 25922, reaching 30%. Regarding the studied EO on bacterial strains only and omitting the exceptionally high 30% value, MBC did not exceed 2%. For yeast strains, the highest MBC values were slightly higher, reaching 4% for thymol (TH) on both *C. albicans* ATCC^®^ 90028 and *N. glabratus* ATCC^®^ 15545.

#### Antibiofilm Activity of Selected EOs Against Bacterial and Yeast Biofilm

The antibiofilm activity of the selected EOs against bacterial strains is presented in Figure 6. In general, the *S. aureus* ATCC^®^ 25923 biofilm was more resistant to the studied EOs than the *E. coli* ATCC^®^ 25922 biofilm. On average, the MBEC_50_ and the MBEC_90_ values were, respectively, 2.5-fold and 5.5-fold higher for *S. aureus*, indicating higher resistance. The lowest MBEC_50_ and MBEC_90_ values against *S. aureus* were observed for CL-MIX and CA, where, for both, the first value reached 0.25% concentration, while the latter values were obtained at 0.5% and 1% concentrations, respectively. High antibiofilm activity of CA was also observed against *E. coli*, with the lowest of the studied concentrations (0.125%) resulting in the acquisition of the MBEC_90_ value. Thus, for CA against *E. coli*, the MBEC_50_ value was not obtained; the value is lower than the 0.125% concentration. The highest MBEC_90_ value was obtained for TTO against *S. aureus*, reaching 4% concentration. For CL-MIX against *E. coli*, the MBEC_90_ value was not obtained (>4%). The comparison of the MBC, MBEC_50_ and MBEC_90_ values is presented in Figure 7.

The antibiofilm activity of the selected EOs against yeast strains is presented in Figure 8. Among all the studied EOs against *N. glabratus* ATCC^®^ 15545, an MBEC_50_ value was established only for CB. For CL-MIX, CA and CV against *N. glabratus*, the MBEC_90_ value reached 0.125% concentration. For CB, the MBEC_90_ was 4-fold higher and, for TTO, both the MBEC_50_ and the MBEC_90_ values exceeded the tested concentration range (>4%).

*C. albicans* ATCC^®^ 90028 biofilm proved higher resistance against the tested EOs. The lowest MBEC_50_, at the 0.125% concentration, was obtained for CB. The highest MBEC_50_ was obtained for TTO at the 0.5% concentration. MBEC_90_ values ranged from 0.25% for CB to 2% for CV. The comparison of MFC and MBEC_50_ values is presented in Figure 9.

### 3.3. Essential Oil Cytotoxicity Assessment

The cut-off value was set at 70% of cell viability; values below that were understood as representing a significant cytotoxic effect. The red, dotted line represents this value on each graph (Figure 10, Figure 11, Figure 12 and Figure 13). In Figure 10, we observed that the cytotoxic effect among four representatives of the citronellol group was demonstrated for concentrations of 0.016% and higher, which was much lower than the concentration needed for the biocidal effect in each. MG-63 cells in CIO-MIX at 0.004% did not reach 50% cell viability, and those in CIO-JT at 0.004% could not reach 40% cell viability. At this concentration, no biocidal activity against microorganisms was observed.

In Figure 11, representing the tea tree group, the concentration at which the cell viability reached 70% was slightly higher (from 0.063%). For the MG-63 cell line, TTO and T4 at a concentration of 0.004% remained toxic. FaDu cell-line viability reached values above 70% only at 0.004% concentrations for TTO and T4.

Figure 12 represents the cinnamon group, where CO, CB, EUG and CA proved the most effective against microorganisms. A strong cytotoxic effect was observed in the studied cell lines. CA exhibited high cytotoxicity, with only the A549 cell line reaching the cut-off value at the lowest studied concentration of 0.004%. Such an effect was much less pronounced for CO, with the cytotoxic effect beginning to diminish at 0.016%.

For TO from the thyme group, only the lowest studied concentration showed viability greater than 70% in all studied cell lines (see Figure 13). The cytotoxic effect started to diminish at 0.016%. CV proved to have a much more pronounced effect, with the cytotoxicity diminishing at 0.008%, and, similarly to TO, only the lowest concentration allowed the cell lines to reach the cut-off value.

### 3.4. Selectivity Index

To display the potential therapeutic window, SI has been calculated. Figure 14 reports the obtained results.

The obtained SI values ranged from 0.004 to 1. The highest value was obtained for TTO against *E. coli* in the biofilm (MBEC_50_) on the A549 cell line. The second highest SI values (SI = 0.5) were obtained for TH against *E. coli* and *S. aureus* in planktonic form (MBC) on the MG-63 and FaDu cell lines, and for T4 against *E. coli* and *N. glabratus* in planktonic form (MBC/MFC) on the A549 cell line. In the biofilm, an SI value of 0.5 was also recorded for TTO against *C. albicans* (MBEC_50_) on the A549 cell line. Similar but slightly lower values (SI = 0.48) were obtained for CL-MIX against *E. coli* and *C. albicans* (MBEC_50_), and against *N. glabratus* (MBEC_90_) on the A549 cell line. The remaining SI values were significantly lower (Figure 14; Supplementary Table S1).

## 4. Discussion

The initial hypothesis assumed that selected EOs would exhibit strong biocidal activity against both bacteria and yeast strains while maintaining non-significant cytotoxicity towards human cell lines. The results partially confirmed this hypothesis; although the biocidal effect was pronounced, substantial cytotoxicity was observed in the studied cell lines. Despite the partial confirmation, the hypothesis is promising, as there are alternative methods of EO administration that directly address cytotoxicity. In the following sections, our findings are discussed in the context of the recent literature, alternative EO delivery strategies, and the underlying mechanism of biological activity.

Our results showed strong inhibition of microbial growth, which is in line with the results obtained by other authors [8,9,10,11,12]. The citronellol group showed a stronger biocidal effect towards *S. aureus* than *E. coli* (Figure 5). The higher resistance of Gram-negative bacteria to EOs from the citronella group, as well as EOs in general, is well supported in the literature [13,14,15,16]. The pronounced difference in MBC values between *E. coli* and *S. aureus* observed for CiO-JT is most likely attributable to its nature as a whole-plant EO rather than an isolated and concentrated constituent. The high biocidal efficacy of EOs from the citronellol group on *C. albicans* is in line with the literature [17,18]. To the best of the authors’ knowledge, no data are available for comparison that might explain the greater effect observed against *N. glabratus*.

Other groups of EOs did not show such unanimous differences in bactericidal activity between *E. coli* and *S. aureus*. The lowest MBC/MFC values, and thus the greatest biocidal activity, were reported for the cinnamon group, especially CA and CB. These findings are well substantiated by the literature [19,20].

When the biocidal activity against planktonic and biofilm forms of whole EOs was compared with that of their isolated components, distinct patterns emerged.

For CB and CO, biocidal activity closely reflected that of their major constituents. CB showed the lowest MBC values among all the tested EOs, which corresponded well with the high potency of CA, its major component. The MBEC_50_ values for CB and CA were comparable to the MBC/MFC values, while the MBEC_90 values_ were mostly higher than the MBC/MFC values. Similarly, the profile of CO paralleled that of EUG, with both the whole oil and the isolated compound exhibiting low MBC/MFC values. The close alignment between the antimicrobial activity of these EOs and their major constituents indicates that their biocidal activity is largely driven by the single molecule, rather than by synergistic interactions within components of the whole EO.

A similar pattern was observed for TTO. Although TTO showed relatively weak antibiofilm activity, its MBC/MFC values were comparable to those of its main component, T4. The slightly higher activity of T4 likely reflects the higher effective concentration of the isolated compound rather than a different mechanism. Notably, as an exception to other strains, TTO exhibited good antibiofilm activity against *C. albicans* (Figure 8). The biological activity of TTO is largely explained by its major constituent (T4) rather than by synergistic interactions within the EO mixture.

CIO-JT displayed a more nuanced pattern. Although the FT-IR assay confirmed the presence of spectra corresponding to citronellal, geraniol, and citronellol in the whole EO, the antimicrobial activity of CIO-JT was broadly comparable to that of citronellol (CL-MIX, CL, β-CL). This indicates that citronellol reflects the intrinsic antimicrobial activity of CIO-JT. A pronounced discrepancy was observed only for *E. coli*, where CIO-JT and CL-MIX exhibited elevated values. Strong activity of CL-MIX against yeast biofilm and moderate activity against bacterial biofilm further substantiates the role of citronellol as the constituent responsible for CIO-JT antimicrobial activity.

The thyme group displayed a mixed pattern. For bacterial strains, TO, TH and CV showed very similar MBC values, indicating that there is a lack of synergy between TH and CV when they are present together as the constituents of TO. When comparing bacterial and yeast strains, TH showed the most pronounced difference in biocidal activity among all the studied EOs, with bacterial strains being susceptible to a concentration 8-fold lower than those required for yeasts (Figure 5). A similar 8-fold difference was previously reported [21]. For TO and CV, the MFC values remained relatively low, whereas for TH the MFC values were significantly higher (Figure 5), indicating that CV plays a more important role in antifungal activity. This phenomenon was further supported by the MBEC assay (Figure 8) and has been demonstrated in the literature [22].

The combined results of the MBC/MFC and the MBEC_50/90_ assays, together with the FT-IR data, provide a basis to infer that, for most of the EOs studied, the major constituents are primarily responsible for the observed biocidal activity.

In most cases, the MBEC_90_ values revealed that the biofilm form required a higher concentration of EOs than those needed to achieve the MBC/MFC values (Figure 7 and Figure 9), reflecting the markedly reduced susceptibility associated with biofilm. Nguyen et al. showed that tea tree oil at 0.5% and 1% concentrations effectively eliminates biofilm persister cells of *E. coli* and S. epidermidis, respectively [23]. These results are promising, yet our results showed that TTO is much less effective in eradicating biofilm forms across the tested EOs. Notably, TTO presented the weakest antibiofilm activity towards *S. aureus* and yeast strains (Figure 6 and Figure 8). For CA at the concentration of 3 mM, Olszewska et al. observed a more than 60% reduction in biofilm metabolic activity on *E. coli* biofilm, which contrasts with our values. The MBEC_90_ for CA on *E. coli* biofilm was obtained at a concentration more than threefold higher (0.125%, ~10 mM) [24]. Among the yeast strains, CB also showed high antibiofilm activity, which aligns with findings from other studies [25]. CV showed high biocidal activity against both planktonic and biofilm forms of the *C. albicans* and *N. glabratus* (Figure 5 and Figure 8). Miranda-Cadena et al. showed similar results, also identifying CA as a potent antifungal [26]. Interestingly, the substantial difference observed between CV and TH in our MFC assay (Figure 5) is not supported by the abovementioned study.

Cytotoxicity assessment performed on A549, M-G63 and FaDu cell lines revealed a largely consistent, concentration-dependent pattern across the tested EOs. The concentrations required to achieve the MBC or MFC values were simultaneously cytotoxic to the cell lines (Figure 10, Figure 11, Figure 12 and Figure 13). Niksic et al. studied the effect of TO on cancerous cell lines and similarly identified dose-dependent cytotoxicity [27]. TH and CV in the A549 cell line posed as an exception to a concentration-dependent pattern in our study (Figure 13). Other authors did not observe such a phenomenon [28,29]. A549 and MG-63 treated with CO exhibited a U-shaped viability pattern, which has not been reported in the literature [30,31].

Importantly, none of the calculated SI values exceeded 1, indicating that the concentrations required to achieve antimicrobial activity were equal to or higher than those associated with cytotoxic effects. In general, SI values below 1 indicate poor selectivity, meaning that a compound affects mammalian cells at concentrations lower or similar to those needed to inhibit microbial growth. Conversely, SI values above 1 are typically interpreted as evidence of a favorable therapeutic window, where antimicrobial activity occurs at concentrations that remain non-cytotoxic to host cells. In our study, SI values >1 were not observed, thus indicating that the tested essential oils exhibit limited selectivity towards microbial cells and that their antimicrobial potential is constrained by concomitant toxicity to mammalian cells. Consequently, direct therapeutic application of these concentrations may be challenging, and future studies should focus on alternative delivery systems, combinations with conventional antimicrobials, or strategies aimed at improving selectivity.

As EOs are studied for potential applications in cancer treatment, their cytotoxicity to human cells represents a critical aspect that requires thorough evaluation. The indicated antimicrobial mechanisms may affect human cells in a similar manner, as they share a lipid-bilayer structure. The abovementioned effect of thymol on fungi may similarly induce the apoptotic cascade via mitochondria [29]. Additionally, it has been indicated that petitgrain EO may affect cell-cycle regulation, causing cell-cycle arrest in FaDu cell lines [32].

Encapsulation of the EOs in liposomes or chitosan nanoparticles can improve biocompatibility by reducing the cytotoxic effects [33,34]. Such an approach has also proven to enhance EO activity against microorganisms [35]. Granata et al. studied the influence of oregano and thyme EOs on *S. aureus*, *E. coli* and Listeria monocytogenes. When EOs were encapsulated in chitosan nanoparticles, the MIC and MBC values dropped significantly [36]. Vitali et al. showed the efficacy of CV encapsulated in chitosan nanoparticles against yeast strains [37]. Encapsulation can also be beneficial due to reduced cytotoxicity [38]. Emtiazi et al. studied the Achillea millefolium EOs and indicated both lower cytotoxicity and higher antibacterial activity when encapsulated [39]. Chitosan microparticles loaded with EOs presented their effectiveness against multispecies biofilm of *C. albicans* and *S. mutans* and simultaneously reported reduced cytotoxicity [40].

Another approach is the use of EOs as an adjuvant in conventional antibiotic therapies. Iseppi et al. studied the interactions between EOs and oxacillin used against methicillin-resistant *S. aureus* (MRSA). Synergy between the studied EOs and antibiotics has been observed, supporting the effectiveness of this combination [41]. In such an approach, synergy enables both compounds to be used at lower concentrations, which is beneficial for EO cytotoxicity. Synergy between antibiotics and EOs in nanoemulsions is a promising approach against multidrug-resistant bacteria [42,43].

A limitation of this study is the lack of the minimum inhibitory concentration (MIC) assessment. The turbidity of the EO solution makes the MIC evaluation prone to errors or even impossible in some cases. Another limitation is that not all the EOs were studied for biofilm inhibition and eradication. A further methodological constraint is that antibiofilm activity was assessed using a test based on metabolic activity, without supplementary microscopic analysis. Although this approach was appropriate for quantitatively determining the MBEC_50_ and MBEC_90_ values, microscopic methods could have provided additional information on the biofilm’s response to essential oils. In particular, confocal scanning microscopy using staining that distinguishes between live and dead cells would allow for the acquisition of data on biofilm architecture and the spatial distribution of live and damaged cells, while scanning electron microscopy would reveal morphological and structural changes. Such supplementary analyses should be considered in future studies. Finally, the use of cancerous cell lines, which can differ in sensitivity to toxic compounds when compared to non-transformed cells, limits further extrapolation of the results. The main advantage of this study is its clear indication of the cytotoxic effect of the studied compounds in human cell lines at concentrations effective against microorganisms.

Taken together, these findings highlight both the therapeutic potential and the inherent limitations of essential oils. While their broad-spectrum antimicrobial activity is well supported by our results and the available literature, the parallel cytotoxic effects observed at biocidal concentrations underscore the need for careful optimization of delivery strategies and formulation approaches. Future studies should therefore focus on improving selectivity, reducing toxicity, and evaluating EO-based therapies in more physiologically relevant models. Such efforts may pave the way toward the safe and effective clinical use of essential oils as complementary antimicrobial agents.

## 5. Conclusions

This study showed strong biocidal activity of selected EOs from citronellol, tea tree, cinnamon and thyme groups against *S. aureus*, *E. coli*, *C. albicans,* and *N. glabratus* in both planktonic and biofilm form. The highest antimicrobial activity was observed in the cinnamon group, while the thyme group displayed the greatest variability in antimicrobial potential across the studied species. Biocidal activity of the EOs and their major components was similar in most studied groups, except for the thyme group, indicating a lack of synergism between studied compounds in each group. Higher MBEC values than the MBC/MFC values confirmed the reduced susceptibility associated with biofilm. A critical limitation identified in this study is the pronounced cytotoxicity of the tested EOs toward human cell lines. The concentrations required to achieve antimicrobial effects exceeded the cytotoxicity threshold, suggesting that the potential therapeutic use of the unmodified EOs is questionable. These findings indicate that EOs should be regarded as potential adjuvants rather than as standalone therapeutic agents. Future research should focus on the development of promising delivery systems, such as nanoemulsions or encapsulation strategies. Further evaluation of synergistic interactions of EOs with conventional antimicrobials should be undertaken. It is only by improving their safety profile that EOs and their major components can be translated into clinically applicable antimicrobial interventions.

## Figures and Tables

**Figure 1:**
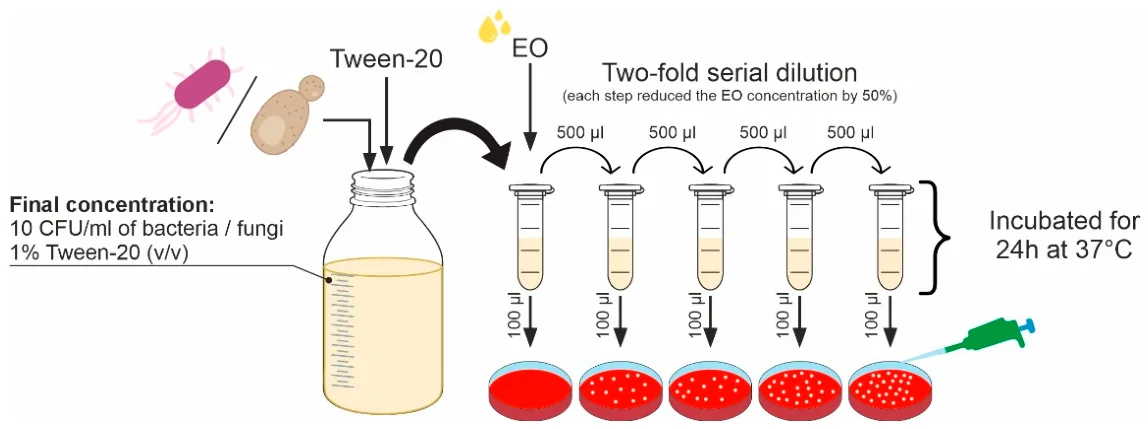
Determination of the MBC and MFC concentrations of EOs using the broth microdilution method.

**Figure 2:**
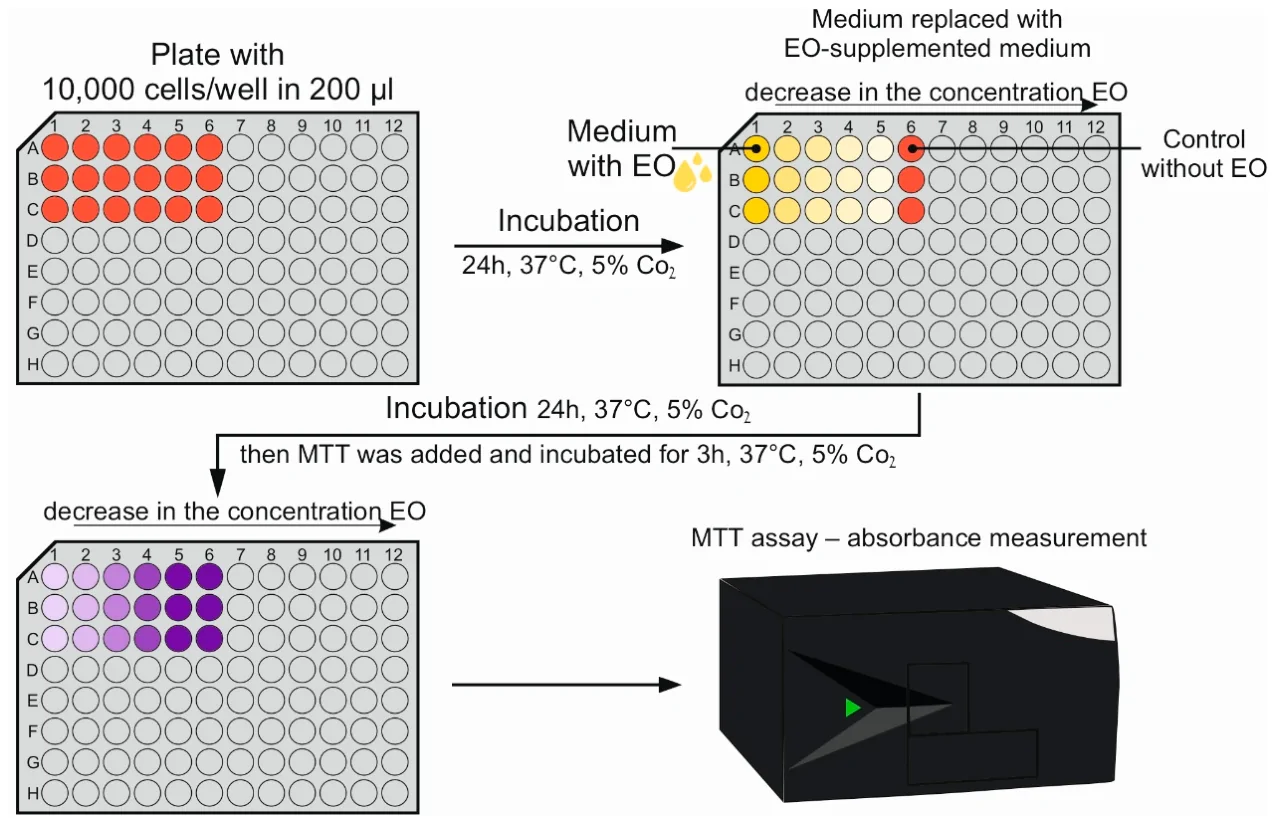
Determination of cell viability after exposure to EOs using the MTT assay.

**Figure 3:**
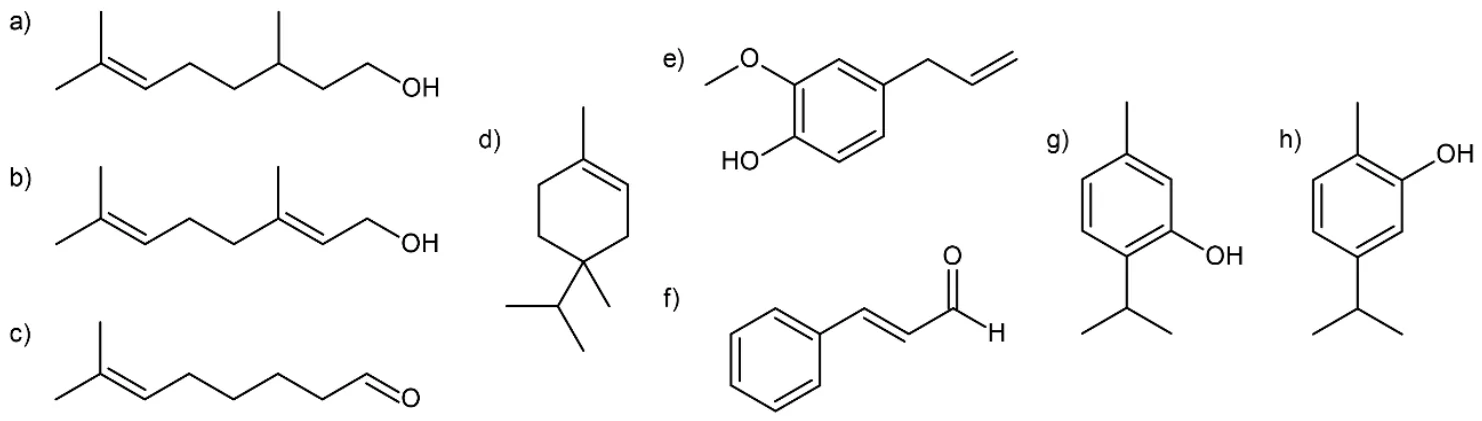
The structure of the studied essential oils components: (**a**) citronellol, (**b**) geraniol, (**c**) citronellal, (**d**) terpinen-4-ol, (**e**) eugenol, (**f**) cinnamaldehyde, (**g**) thymol, (**h**) carvacrol.

**Figure 4:**
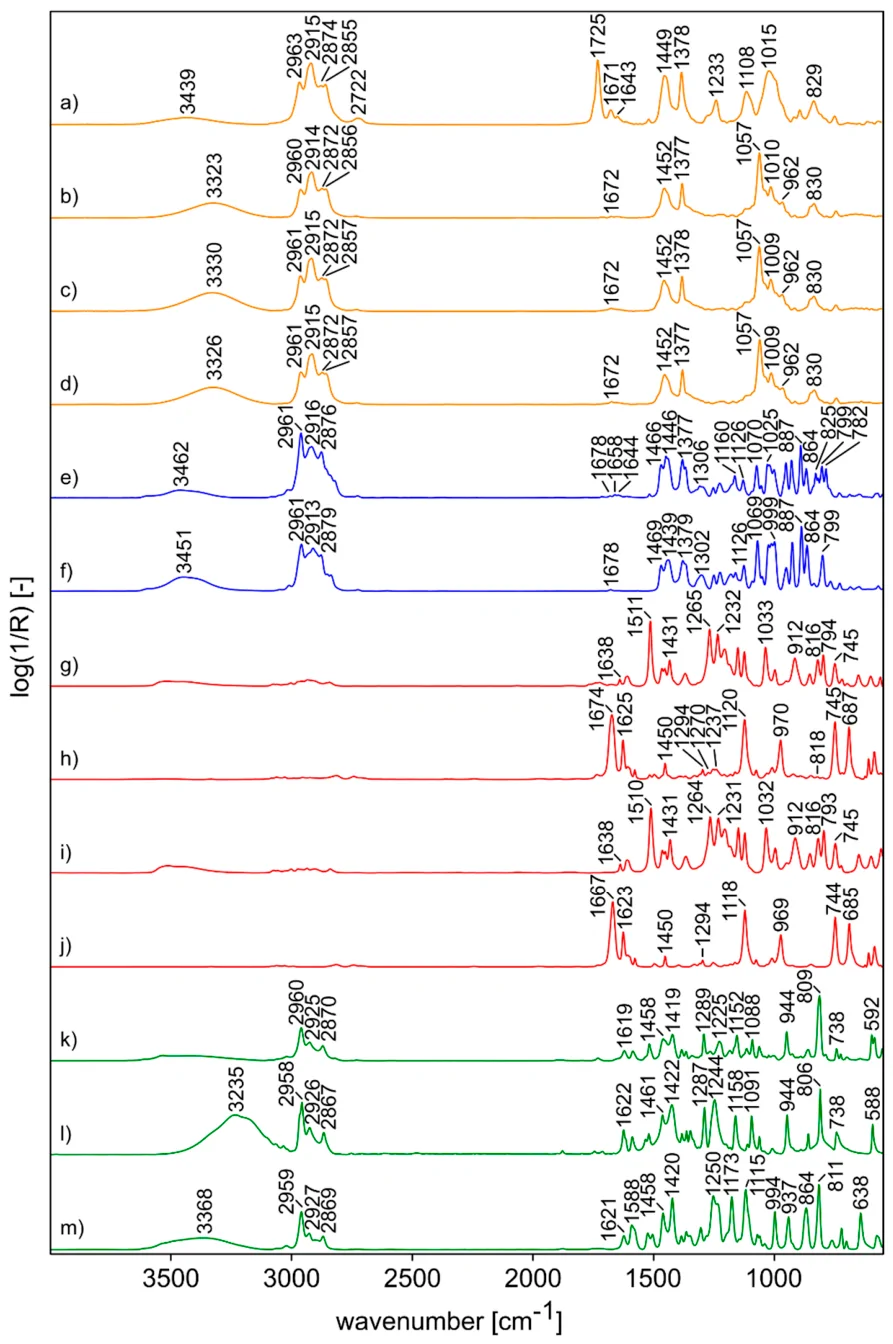
FT-IR spectra for the analyzed essential oils and their components: (**a**) Java-type citronella oil, (**b**) citronellol (mixture of isomers), (**c**) citronellol, (**d**) β-citronellol, (**e**) tea tree oil, (**f**) terpinen-4-ol, (**g**) cinnamon leaf oil, (**h**) cinnamon bark oil, (**i**) eugenol, (**j**) cinnamaldehyde, (**k**) thyme oil, (**l**) thymol, (**m**) carvacrol. For clarity, only the most characteristic bands have been assigned. Different colors were used to identify the spectra of the substances from specific groups—orange for citronellol, blue for tea tree, red for cinnamon, and green for thyme.

**Figure 5:**
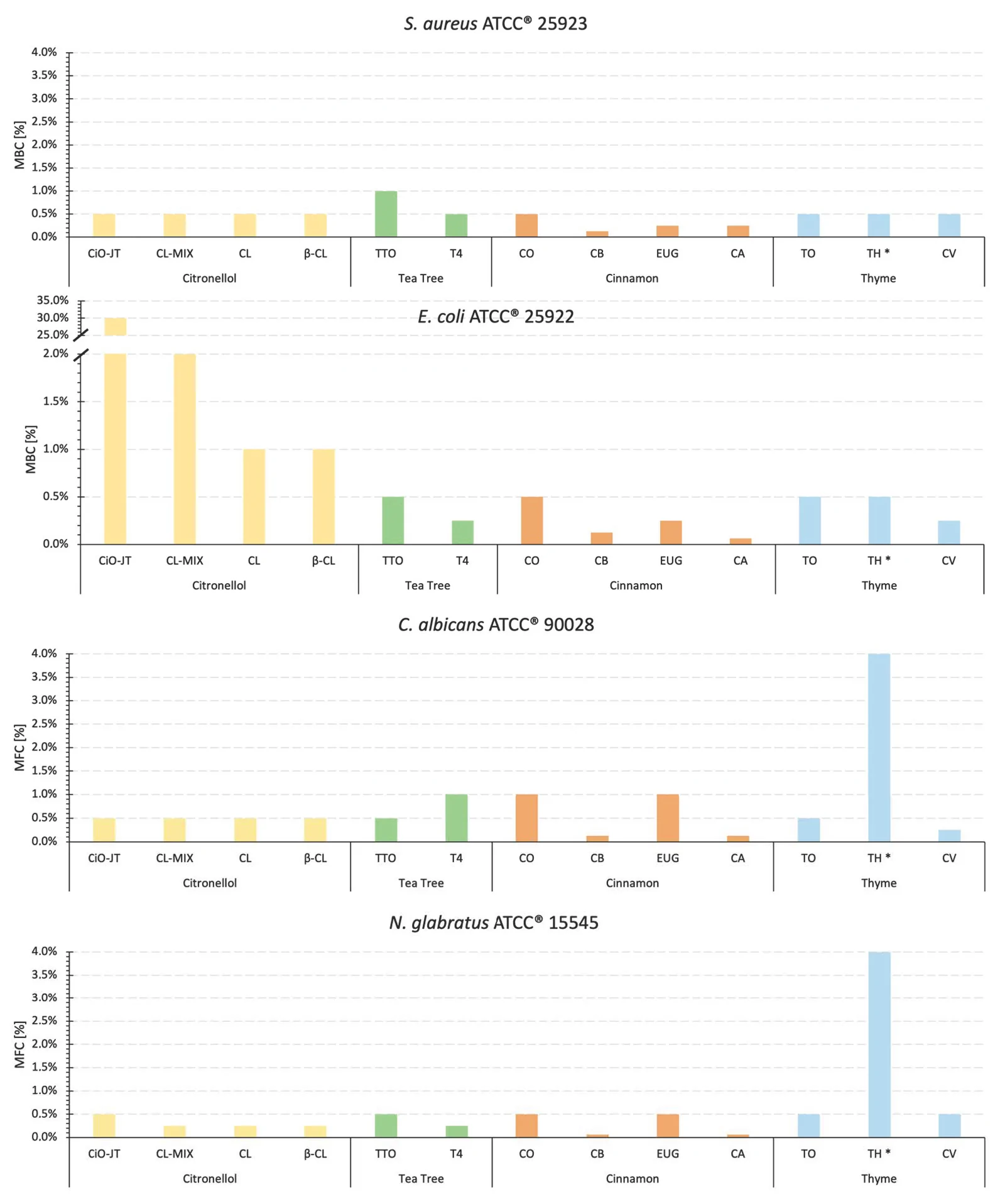
Minimum bactericidal concentration (MBC) of studied EOs on selected bacterial and minimum fungicidal concentration (MFC) yeast strains. Legend: CiO-JT—Java-Type Citronella Oil; CL-MIX—Citronellol mixture of isomers; CL—Citronellol; β-CL—β-Citronellol; TTO—Tea Tree oil; T4—terpinen-4-ol; CO—Cinnamon leaf oil; CB—Cinnamon bark oil; EUG—Eugenol; CA—Cinnamaldehyde; TO—Thyme oil; TH—Thymol; CV—Carvacrol; *—mass/volume percentage, where 100% is the maximum solubility of the compound.

**Figure 6:**
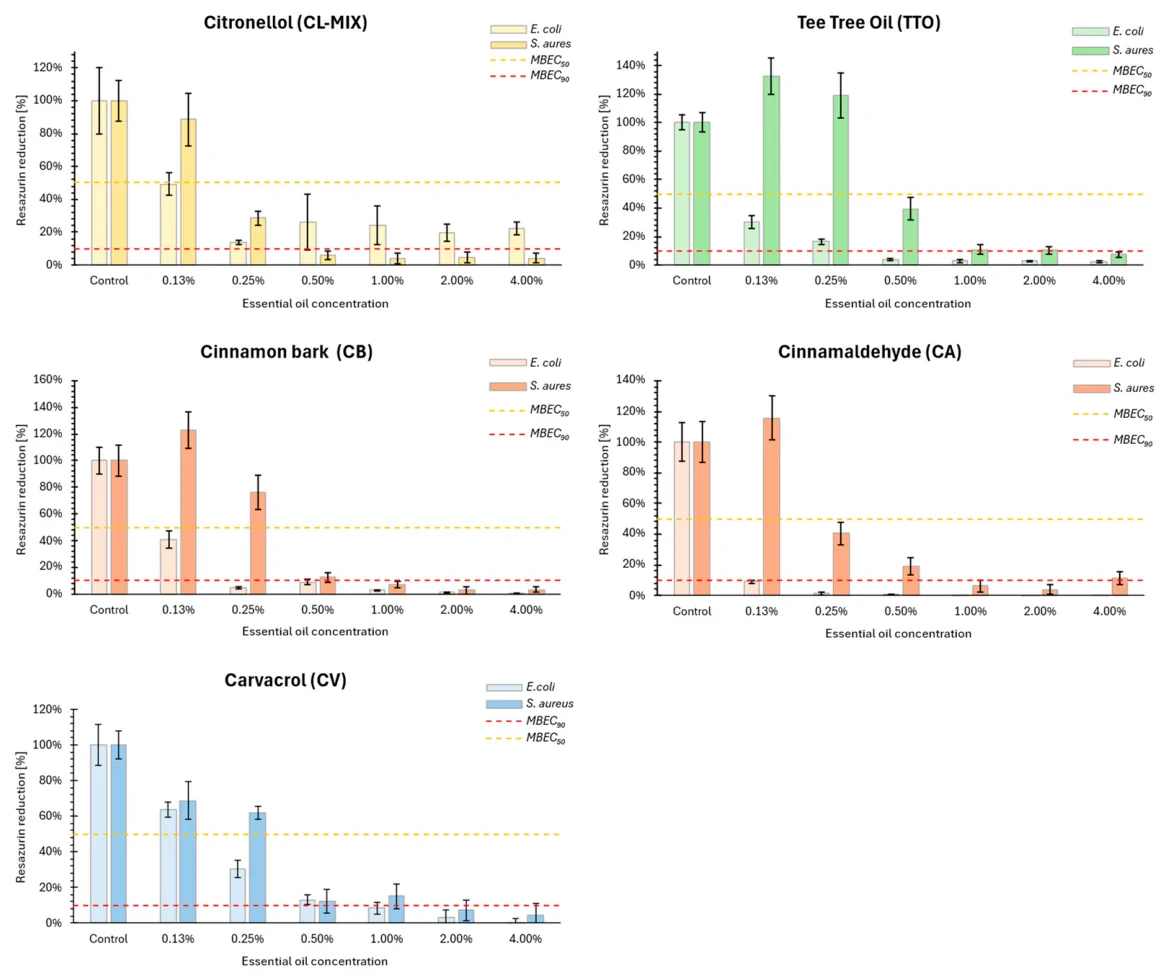
MBEC_50_ and MBEC_90_ of selected EOs on bacterial strains. Legend: CL-MIX—Citronellol mixture of isomers; TTO—Tea Tree oil; CB—Cinnamon bark oil; CA—Cinnamaldehyde; CV—Carvacrol.

**Figure 7:**
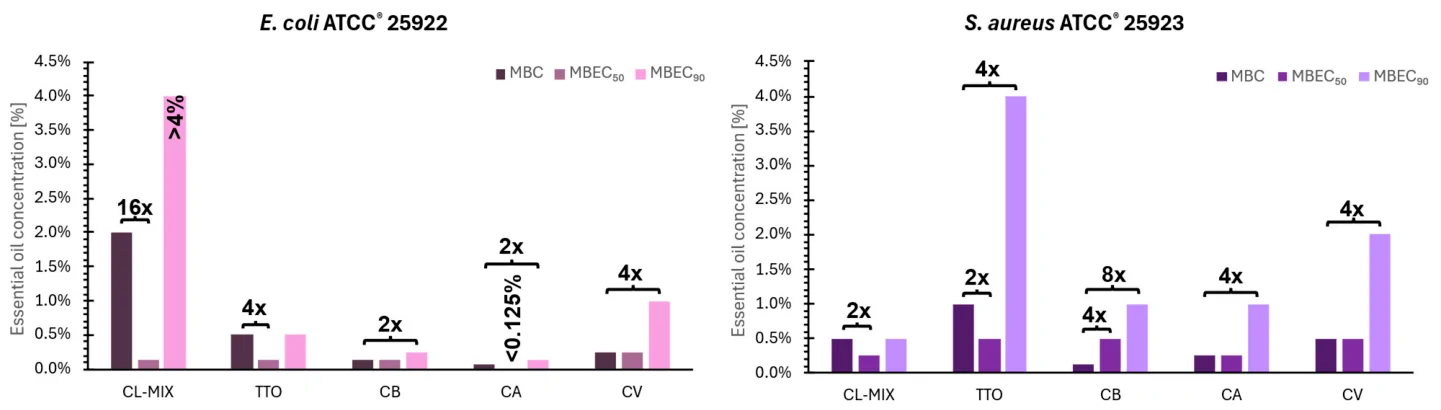
Comparison of MBC, MBEC_50_ and MBEC_90_ values for *E. coli* ATCC^®^ 25922 and *S. aureus* ATCC^®^ 25923. The labels (e.g., 8×) indicate the fold difference between the corresponding MBC and MBEC values. >4%/<0.125% indicates that the MBEC value could not be determined.

**Figure 8:**
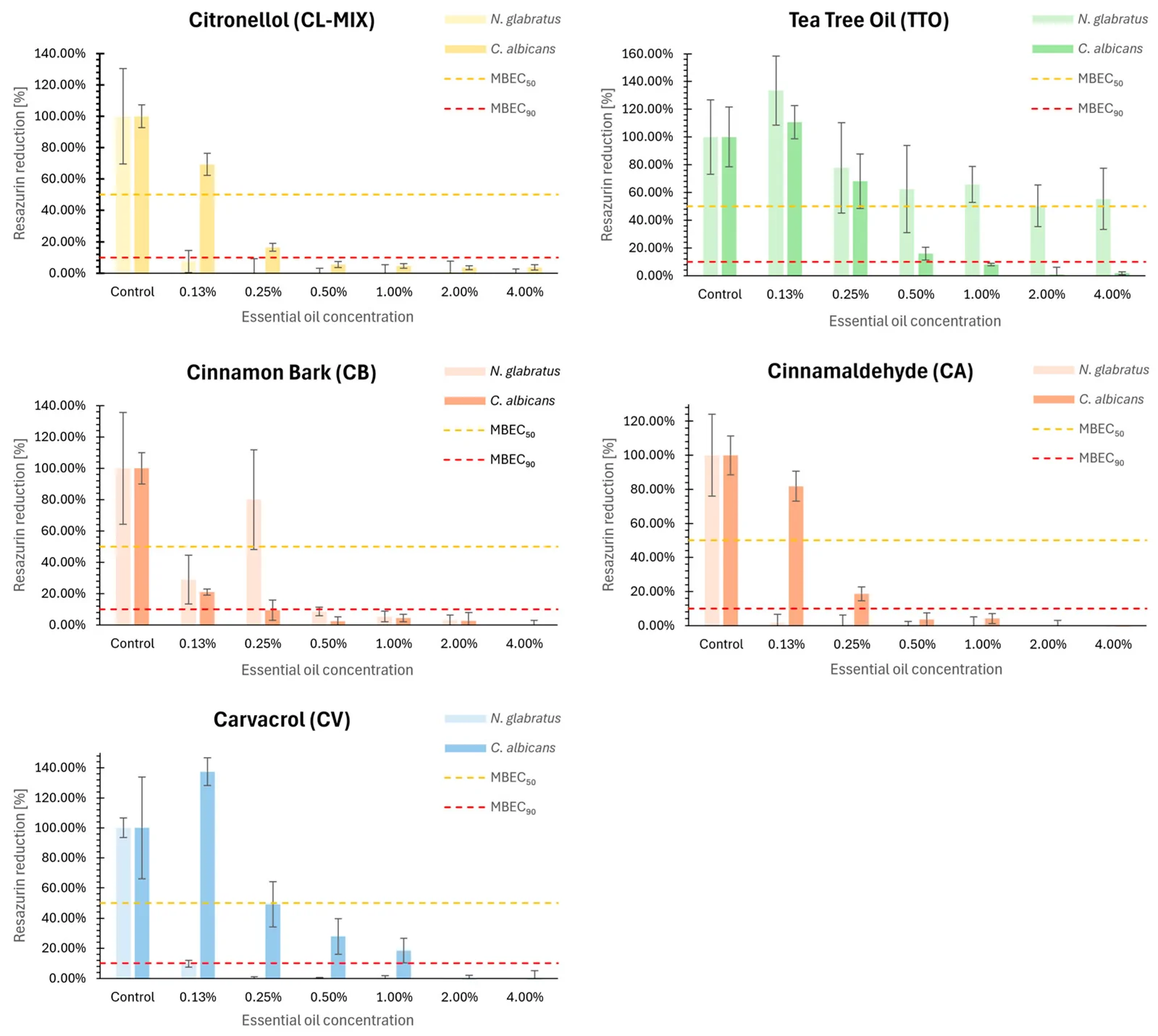
MBEC_50_ and MBEC_90_ of selected EOs on yeast *C. albicans* ATCC^®^ 90028, and *N. glabratus* ATCC^®^ 15545. Legend: CL-MIX—Citronellol mixture of isomers; TTO—Tea Tree oil; CB—Cinnamon bark oil; CA—Cinnamaldehyde; CV—Carvacrol.

**Figure 9:**
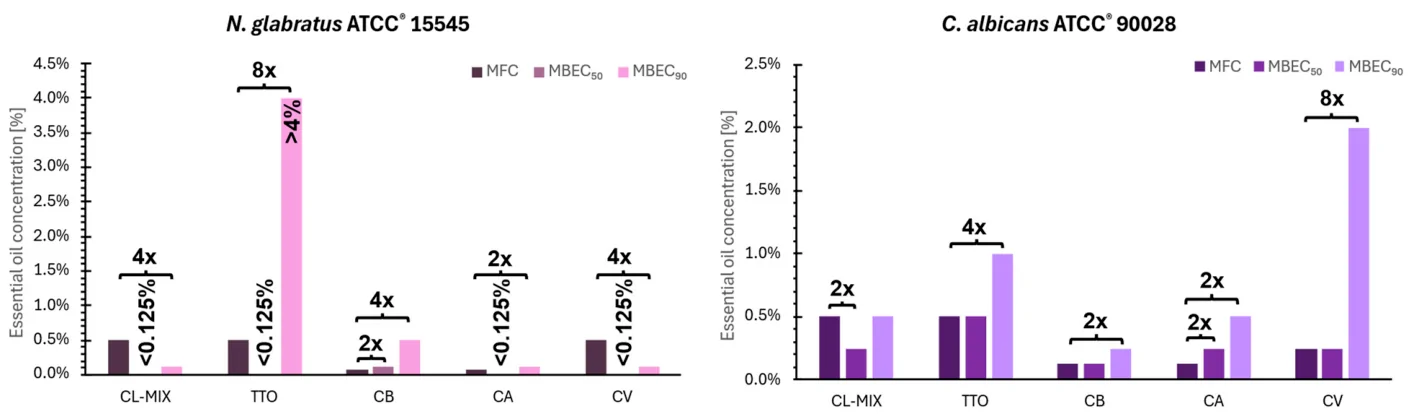
Comparison of MBC, MBEC_50_ and MBEC_90_ values for *C. albicans* ATCC^®^ 90028 and *N. glabratus* ATCC^®^ 15545. The labels (e.g., 8×) indicate the fold difference between the corresponding MBC and MBEC values. >4%/<0.125% indicates that the MBEC could not be determined.

**Figure 10:**
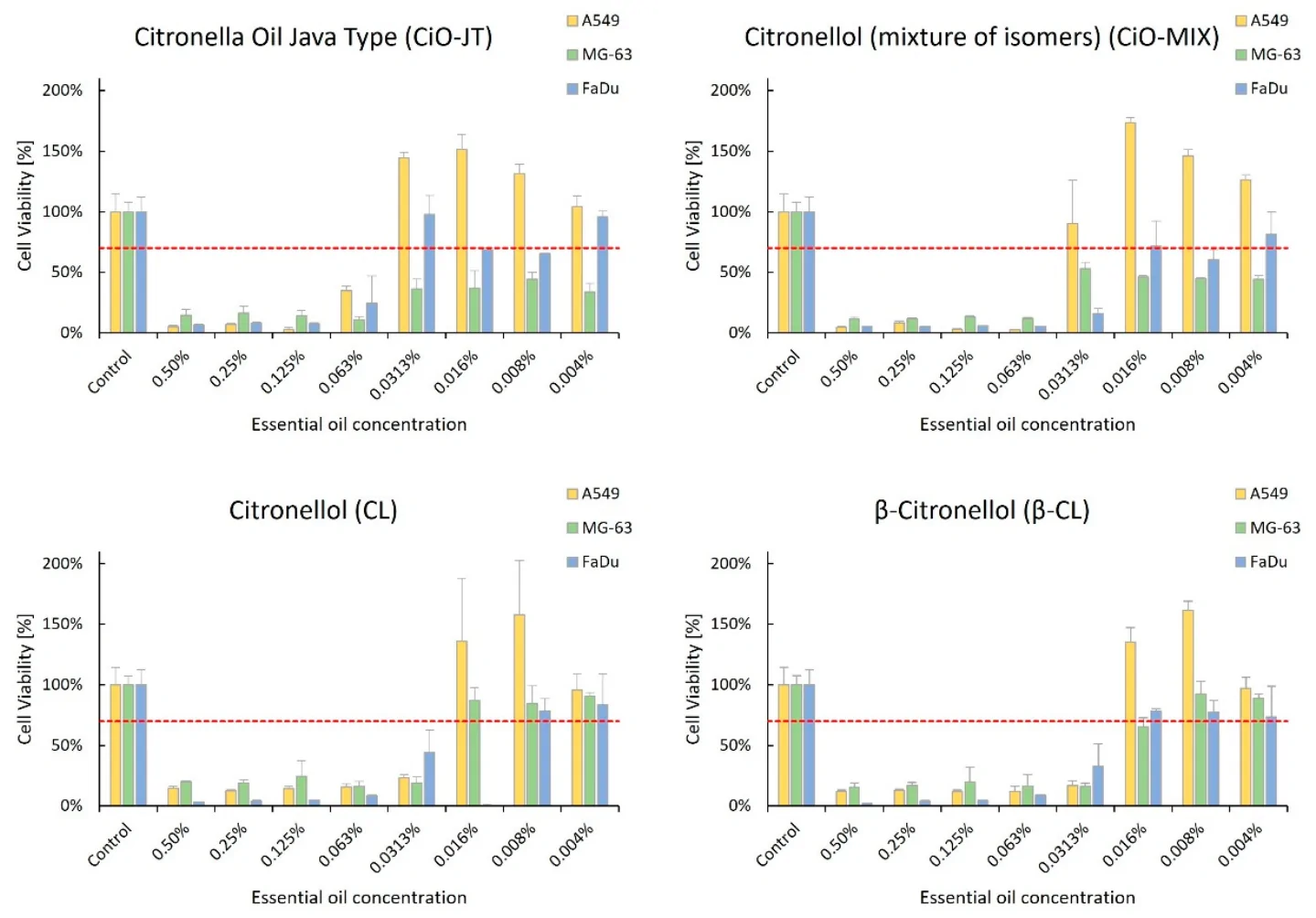
Mean viability values of studied cell lines, A549—yellow, MG-63—green, FaDu—blue, in relation to concentration of EOs from the Citronellol group. The red dotted line indicates viability at the 70% level.

**Figure 11:**
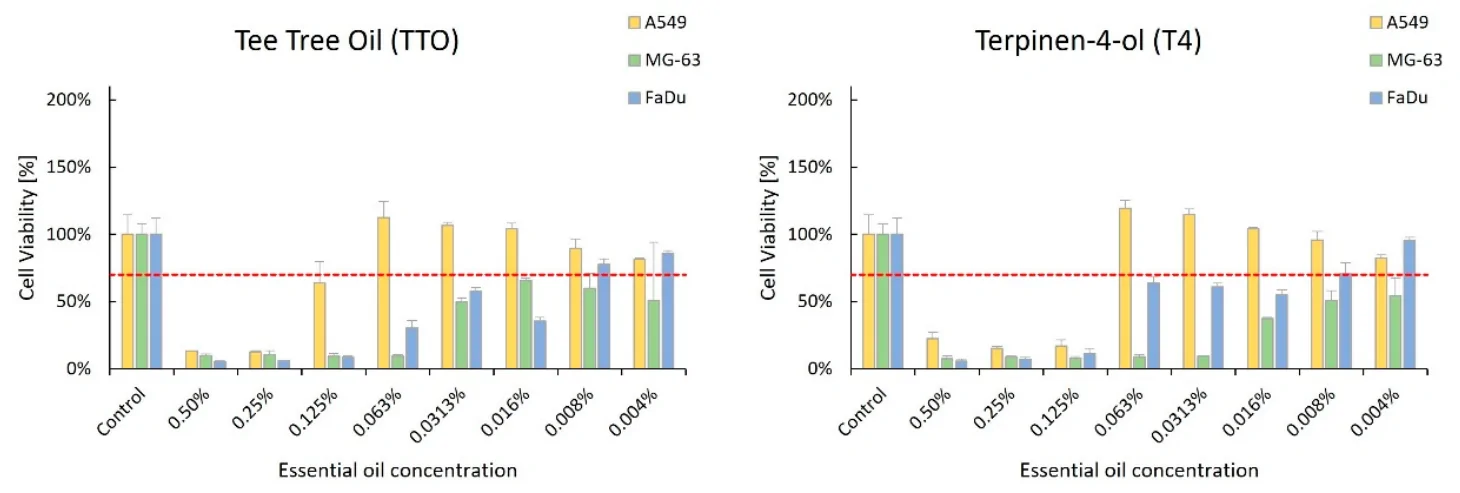
Mean viability values of studied cell lines, A549—yellow, MG-63—green, FaDu—blue, in relation to concentration of EOs from tea tree group. The red dotted line indicates viability at the 70% level.

**Figure 12:**
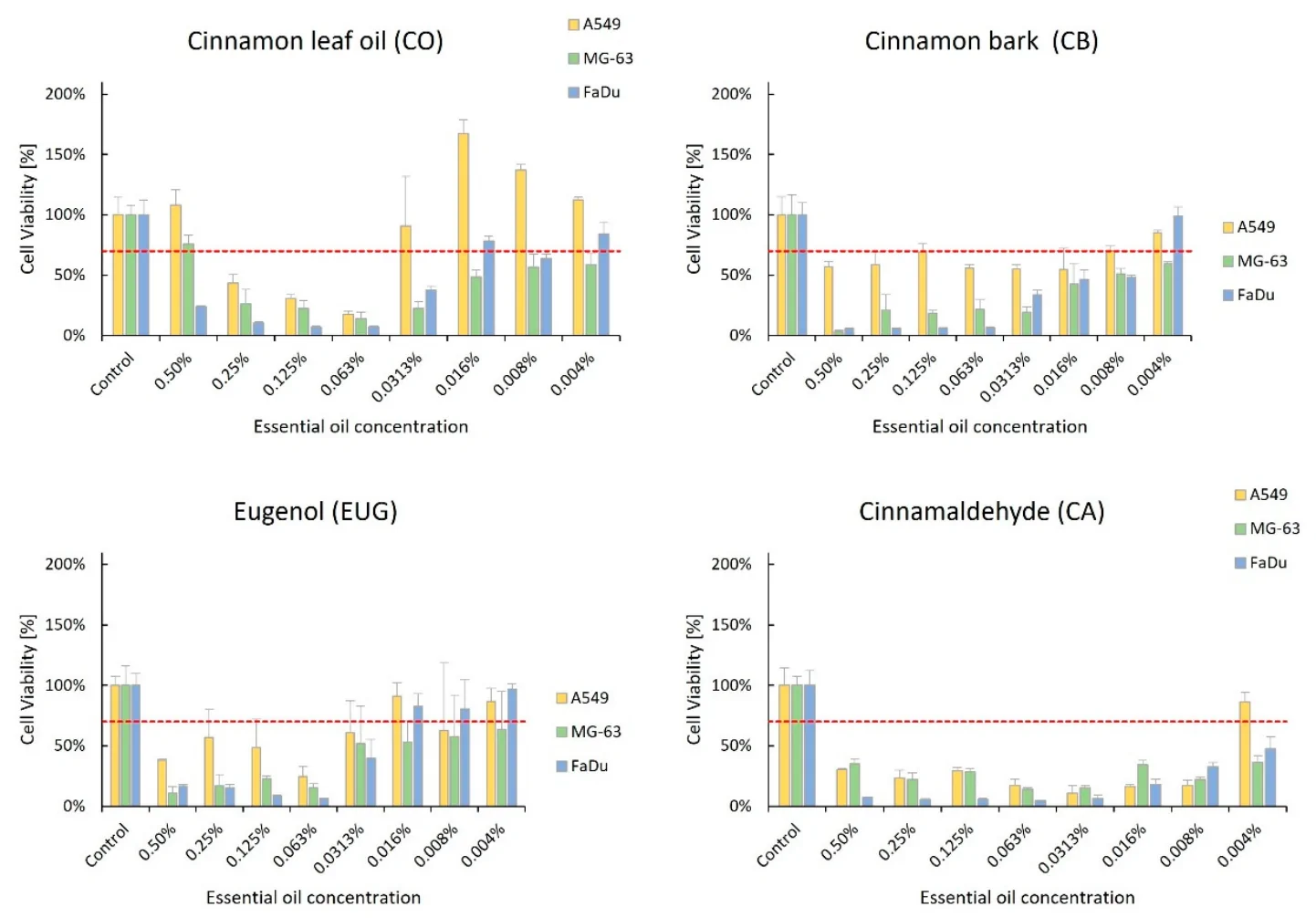
Mean viability values of studied cell lines, A549—yellow, MG-63—green, FaDu—blue, in relation to concentration of EO from Cinnamon group. The red dotted line indicates viability at the 70% level.

**Figure 13:**
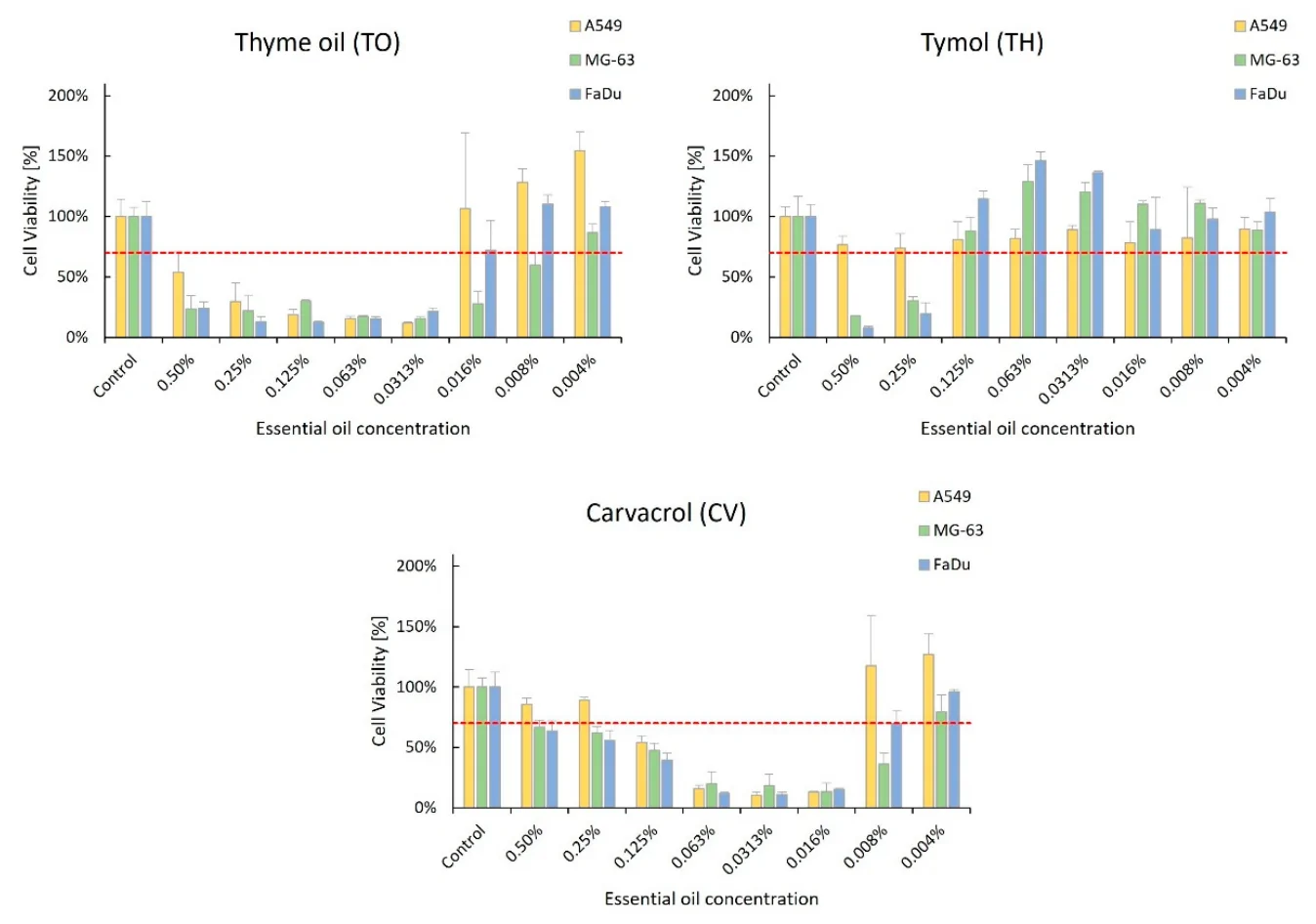
Mean viability values of studied cell lines, A549—yellow, MG-63—green, FaDu—blue, in relation to concentration of EO from the Thyme group. The red dotted line indicates viability at the 70% level.

**Figure 14:**
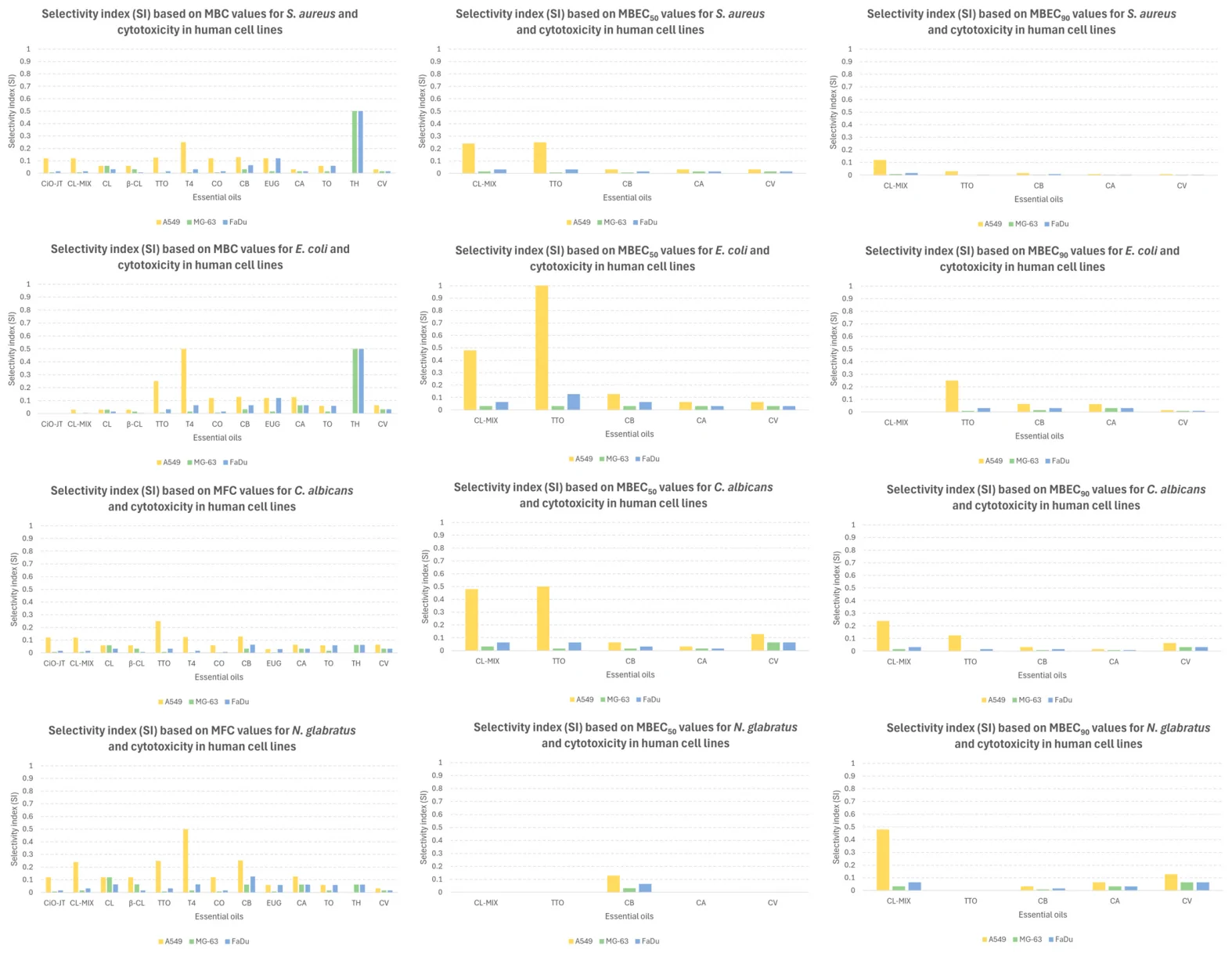
Selectivity index (SI) values for the studied microbial strains and human cell lines. Legend: CiO-JT—Java-Type Citronella Oil; CL-MIX—Citronellol mixture of isomers; CL—Citronellol; β-CL—β Citronellol; TTO—Tea Tree oil; T4—terpinene-4-ol; CO—Cinnamon leaf oil; CB—Cinnamon bark oil; EUG—Eugenol; CA—Cinnamaldehyde; TO—Thyme oil; TH—Thymol; CV—Carvacrol;

**Table 1:** Overview of the tested EOs and their constituents.

Group	Name	Abbreviation	Supplier (Catalog Number)
Citronellol	Java-Type Citronella Oil	CiO-JT	Pol-Aura (Zawroty, Poland) (PA-15-0135)
	Citronellol (mixture of isomers)	CL-MIX	Merck (Rahway, NJ, USA) (W230915-100G-K)
	Citronellol	CL	Merck (W230901-1KG-K)
	β-Citronellol	β-CL	Merck (C83201-100G)
Tea Tree	Tea Tree oil	TTO	Pol-Aura (PA-03-8936-K)
	Terpinen-4-ol	T4	Merck (11584-25G-F)
Cinnamon	Cinnamon leaf oil	CO	Merck (W229210-1KG-K)
	Cinnamon bark oil	CB	Merck (W229105-250G-K)
	Eugenol	EUG	Merck (E51791-100G)
	Cinnamaldehyde	CA	Merck (W228613-100G-K)
Thyme	Thyme oil	TO	Merck (W306509-1KG-K)
	Thymol	TH	Merck (T0501-100G)
	Carvacrol	CV	Merck (W224511-100G-K)

## Data Availability

The raw and processed data required to reproduce results presented in this publication are available on https://doi.org/10.58032/AGH/IFTQCY (accessed on 21 September 2026).

## Supplementary Materials

The following supporting information can be downloaded at: https://www.mdpi.com/article/10.3390/microorganisms14102196/s1. Table S1: Selectivity index (SI) values of the studied essential oils and their major constituents calculated for selected microbial strains and human cell lines based on MBC/MFC, MBEC_50_, and MBEC_90_ values.

## Abbreviations

The following abbreviations are used in this manuscript:

AMR	Antimicrobial resistance
ATCC	American Type Culture Collection
CA	Cinnamaldehyde
CB	Cinnamon bark oil
CFU	Colony-forming units
CiO-JT	Java-type citronella oil
CL	Citronellol
CL-MIX	Citronellol (mixture of isomers)
CO	Cinnamon leaf oil
CV	Carvacrol
DMSO	Dimethyl sulfoxide
EO	Essential oil
EOs	Essential oils
EUG	Eugenol
MBC	Minimum bactericidal concentration
MBEC	Minimum biofilm eradication concentration
MFC	Minimum fungicidal concentration
MIC	Minimum inhibitory concentration
MTT	3-(4,5-dimethylthiazol-2-yl)-2,5-diphenyltetrazolium bromide
SB	Sabouraud broth
SI	Selectivity index
T4	Terpinen-4-ol
TH	Thymol
TO	Thyme oil
TSB	Tryptic soy broth
TTO	Tea tree oil
β-CL	β-Citronellol
